# T53. USING ARTIFICIAL INTELLIGENCE PLATFORMS TO ENHANCE STUDY DESIGN IN SCHIZOPHRENIA TRIALS

**DOI:** 10.1093/schbul/sby016.329

**Published:** 2018-04-01

**Authors:** Laura Shafner, Chloe Chah

**Affiliations:** AiCure

## Abstract

**Background:**

Remote patient monitoring is critical in ensuring optimal drug exposure. Between 30–50% of CNS trials fail because patients are not following the assigned protocol. It is estimated that non-adherence based on pharmacokinetic (PK) data is as high as 39% in schizophrenia trials. An AI platform that uses software algorithms on smartphones to visually and automatically confirm medication ingestion has been used extensively in schizophrenia trials, phases I-IV. Aggregated data demonstrate the feasibility of using the technology in patients with schizophrenia – where smartphone ownership is estimated to be well above 50% - and the potential value of enhancing study design through predictive data and statistical power.

**Methods:**

Aggregated data were collected across seven schizophrenia studies; three trials are completed and four are ongoing. Protocols varied by geography, treatment duration, study design, inclusion/exclusion criteria, dosing regimens, and assessment frequency (US and global; six to 52 weeks’ treatment duration, lead-in or washout periods, ages 16–65 years, dosing QD or BID, 1–3 units per dose, inpatient and outpatient). Study subjects used the AI application for each dosing administration. In addition to tracking medication intake, the patient-facing interface also provided automated reminders, alarms, dosing windows, clinic visit scheduling, and protocol-specific dosing instructions. Study teams and sites had access to data, analytics, automated notifications, and intervention dashboards.

**Results:**

So far, 43,340 adherence parameters have been collected in studies with target enrollments of 1,312 subjects with schizophrenia. For randomized subjects who received at least 1 dose of the study drug, cumulative average adherence as measured by the AI platform (visual confirmation of ingestion) across all treatment groups, including placebo, is 83.6%. Adherence, as measured by the percentage of PK samples above the lower limit of quantification (LLOQ), is 91.2%. Between 3.9% and 12.5% of subjects triggered fraudulent activity alerts (intentional misuse of the technology). The average number of site interventions per subject per study was 4.7 (33.8% text messages; 34.7% phone calls; 31.5% in-person clinic visits). Adherence data logged on the AI platform were used in most studies as the primary measure of adherence (for at home and in-clinic dosing) and as the basis to evaluate eligibility criteria for randomization following placebo lead-in periods.

**Discussion:**

Subjects with schizophrenia (stable, acute, positive and negative symptoms, cognitive impairment) treated with antipsychotics demonstrate high rates of adherence using a smartphone-based AI application. Non-adherence based on PK data ranged from 8.3% to 10.4%; a significant reduction from the 39%-50% non-adherence rates observed in clinical trials and real-world settings. Traditional methods to monitor adherence – pill count and self-report methods – are not reliable enough to be of predictive value in lead-in periods, demonstrate poor concordance with PK data, and do not allow sponsors to resolve issues that may affect adherence in real time. Use of the AI platform in schizophrenia studies demonstrates the potential of the technology to enhance data quality, enable the sponsor to estimate the effect of the investigational drug - when used as directed – and improve the likelihood of detecting a signal.

